# The peer-led Honest, Open, Proud program to decrease the impact of mental illness stigma among German military personnel: randomized controlled trial

**DOI:** 10.1007/s00127-025-02960-x

**Published:** 2025-07-22

**Authors:** Nicolas Rüsch, Christian Helms, Jana Hörger, Burkhard Höhle, Hendryk Bernert, Patric Muschner, Carolyn Rose, Patrick W. Corrigan, Nadine Mulfinger, Peter Zimmermann, Gerd-Dieter Willmund

**Affiliations:** 1 Department of Psychiatry II, Ulm University and BKH Günzburg, Ulm/Günzburg, Germany; 2 Center for Military Mental Health, Berlin, Germany; 3 German Bundeswehr Social Service Flensburg, BwDLZ Husum, Germany; 4 Illinois Institute of Technology, Chicago, USA; 5 German Ministry of Defence, EBU III 4, Berlin, Germany

**Keywords:** Military, Stigma, Disclosure, Honest, Open, Proud, Peer support, RCT

## Abstract

**Purpose:**

Due to widespread stigma in the military, many military servicemembers with mental illness struggle with discrimination, self-stigma and decisions whether to disclose their condition. This study had the aim to evaluate the efficacy of Honest, Open, Proud (HOP), a four-session peer-led group program adapted to support military personnel with disclosure decisions and to reduce stigma’s impact, in the German military.

**Methods:**

Ninety-nine active servicemembers with mental illness were randomized to HOP and treatment as usual (TAU) or to TAU alone. The two primary endpoints were stigma stress three weeks after baseline (T1/after HOP for HOP participants) and psychological quality of life six weeks after baseline (T2/after the HOP booster session). This randomized-controlled trial was registered before recruitment onset at ClinicalTrials.gov (NCT03218748).

**Results:**

Compared to the control group, stigma stress decreased significantly among HOP participants at T1 (d = 0.64), while there was no significant effect of HOP on psychological quality of life at T2. HOP had significant positive effects on secondary outcomes at T2: overall quality of life, self-stigma, depressive symptoms, empowerment, well-being, attitudes to disclosure and to help-seeking, and secrecy. Reductions in stigma stress, secrecy, and marginally in overall quality of life (*p* = 0.055), remained significant twelve weeks after baseline (T3).

**Conclusion:**

This trial provides initial evidence that HOP for active military servicemembers is feasible and effective in terms of stigma stress, disclosure decisions, depressive symptoms, quality of life and well-being. HOP may be a valuable addition to mental health services and peer support in the military.

## Introduction

Mental disorders are common among active military servicemembers and veterans [1–3]. In a representative study of German soldiers, the combined 12-month prevalence of depressive disorders, anxiety disorders, and substance use disorders was approximately 20%, while the prevalence of post-traumatic stress disorder (PTSD) was about 2% [4–6]. Low rates of help-seeking cause suffering among military personnel and their families as well as impaired vocational functioning and suicidality [7]. It has been estimated that two thirds of military servicemembers with mental health problems do not seek help [8]. A major barrier to help-seeking is stigma: In a systematic review of quantitative studies, more than 40% of servicemembers endorsed stigma-related concerns due to seeking care, such as being treated differently by officers or feeling weak [8]. This is supported by qualitative studies about anticipated or experienced stigma and career concerns as major barriers to help-seeking [9], which is consistent with previous findings that among people with mental illness in general, military servicemembers are one of the groups most deterred from help-seeking by stigma [10].

The decision whether to disclose a mental illness to others can be difficult for both civilians and military personnel [11]. But in the military culture in particular, many servicemembers with mental health problems struggle with such disclosure decisions [9] due to fear of public stigma and discrimination by others or due to self-stigma and shame [12]. In a previous focus group study in the German military, participants stressed the complexity of disclosure decisions: disclosure can lead to career risks and painful discrimination by colleagues and officers; but non-disclosure is a barrier to help-seeking and often contributes to social isolation and despair [13]. In order to support people with mental illness with their disclosure decisions, Honest, Open, Proud (HOP), formerly known as Coming Out Proud, was developed by Patrick W. Corrigan and colleagues in the US as a peer-led group program [14]. It is not HOP’s aim to make people disclose, but to empower them to weigh pros and cons of disclosure depending on the setting and their individual situation and goals. A meta-analysis based on five randomized-controlled trials showed that HOP in non-military settings led to short and medium-term reductions in stigma-related stress and self-stigma [15]. But to the best of our knowledge HOP has not been evaluated among active military personnel. Colleagues in the US Veterans Administration adapted HOP for veterans and found positive HOP effects in an uncontrolled evaluation [16].

After adapting HOP for the German military, we conducted this RCT with the aim to examine HOP’s efficacy to reduce the impact of mental illness stigma on servicemembers with mental illness in the German military. As two primary outcomes, compared to the control group we expected HOP to reduce stigma stress after the 3-week program (T1); and to improve psychological quality of life after the booster session (T2, 6 weeks after baseline). If one of both primary outcomes showed a significant HOP effect, we would consider the intervention effective. As secondary outcomes, we expected improvements in other stigma- and disclosure-related and clinical outcomes at T1, T2 and follow-up (T3, 12 weeks after baseline).

## Methods

### Trial design and setting

This parallel two-arm RCT compared the efficacy of HOP (and TAU) versus TAU alone. The trial was registered before recruitment onset at https://clinicaltrials.gov/study/NCT03218748 and approved by the ethics committee of Ulm University, Germany (Nr. 245/15). Participants were recruited at inpatient, day clinic and outpatient settings of the Department of Psychiatry, Military Center for Mental Health, Berlin, Germany, from 2017 to 2023. Recruitment was delayed by the covid-19 pandemic and because a psychiatric day clinic of the German military, in which recruitment had been planned, unexpectedly closed soon after recruitment onset.

All participants, in both trial arms, received TAU at the Center for Military Mental Health, Berlin, which included psychiatric-psychotherapeutic care tailored to individual needs. Participants received standard outpatient care (weekly guideline-based individual 50-minute psychotherapy sessions and quarterly psychiatric follow-ups) or inpatient/day clinic care (typically two individual psychotherapy sessions per week, sport psychology once or twice a week, movement-based interventions with nursing staff (3×/week), ergotherapy and art therapy (3×/week), and psychoeducation groups on depression, anxiety, and sleep disorders). A proportion of these participants also received trauma-focused interventions (e.g., EMDR, prolonged exposure, or trauma-focused cognitive behavioral therapy). All therapeutic decisions were made independently of study entry or randomization to HOP or to TAU alone. We did not systematically track the exact therapeutic components each participant received, as our focus was on evaluating the added value of the HOP program.

### Participants

Inclusion criteria were current inpatient, day clinic or outpatient treatment at the Center for Military Mental Health, Berlin, Germany, a mental disorder according to ICD-10 and based on chart diagnoses, age 18 or above, sufficient German language skills to participate in HOP, and written informed consent. Exclusion criteria were an intellectual disability (ICD-10: F7); organic disorder (ICD-10: F0); or diagnosis of only a substance- or alcohol-related disorder (ICD-10: F1), without non-substance related current psychiatric comorbidity, since disclosure of substance use disorders is not a topic specifically discussed in this HOP program and the stigma of substance use disorders differs from mental illness stigma [17].

### Honest, Open, Proud (HOP): program and group facilitators

HOP is a peer-led group program that was developed by Patrick W. Corrigan and colleagues in Chicago, US, based on previous work (hopprogram.org [18]). HOP was translated into German in preparation for a previous non-military trial and is called ‘In Würde zu sich stehen’ in German (in English: To stand up for yourself in dignity [19]). HOP supports participants with their decisions whether and how to disclose their condition in different settings; it is not the aim of HOP to make people disclose, but to support an empowered, personal choice, depending on the participants’ goals and their environment. HOP covers the following topics in four two-hour lessons: (i) weighing the pros and cons of (non-)disclosure; (ii) ways of disclosure, from social avoidance and secrecy to selective or indiscriminate disclosure up to broadcasting one’s experience, and finding good people to disclose to; (iii) ways to tell one’s story, if one decides to do so; and (iv) finally, a booster session on participants’ experiences with (non-)disclosure after the third lesson.

Based on our previous qualitative study on disclosure of mental disorders among members of the German military [13] and on participatory research with a group of military servicemembers with lived experience and an advisory panel, we carefully adapted the HOP version for adults to HOP for active military personnel, keeping the overall structure and content outlined above. During this process we replaced the non-military recovery story in lesson 3 of the general adult HOP version by four stories of German military servicemembers, among them one female, and their lived experience of mental illness, covering different disorders and different military ranks. Each HOP group was facilitated by two peers, i.e. military staff with lived experience of mental illness. All group facilitators had been trained by Nicolas Rüsch and had run HOP practice groups with a fidelity of at least 80% prior to recruitment onset. The peer group facilitators received qualitative feedback after the group sessions how participants had perceived the HOP program and whether it was helpful; a summary of this qualitative information is provided in the Results.

### Fidelity

One research assistant was present in the background of every session and completed the fidelity checklist that was used in previous HOP trials [15]. Fidelity was high with a mean fidelity of 96% for lesson one, 92% for lesson two, 91% for lesson three, and 78% for lesson 4. Mean fidelity across all four sessions was 90%.

### Planned sample size

Prior to this study, there was no data on HOP’s efficacy in the military. Based on a power of 80% to detect an effect on at least one endpoint, adjusted alphas of 0.025, and two primary endpoints with an expected correlation of *r*=−0.3 (unpublished data), 100 participants are sufficient to detect medium effect sizes of d = 0.5 on stigma stress, similar to effects on stigma stress in a previous adult HOP RCT [19], and d = 0.4 on quality of life.

### Randomization

After completing the baseline assessment (T0), participants were randomly assigned to the intervention (HOP and treatment as usual/TAU) or control group (TAU alone). TAU consisted of inpatient, day clinic or outpatient psychiatric-psychotherapeutic mental health care in the Department of Military Mental Health, Berlin, Germany. The randomization lists and closed envelopes were generated by the Institute of Epidemiology and Medical Biometry, University of Ulm, Germany. The trial was started using a 1:1-randomization; due to the very slow recruitment and the consequent difficulty to fill new HOP groups in time before interested potential participants who waited for the start of a HOP group were discharged, exacerbated by the Covid-19 pandemic, the randomization was changed to a 2(HOP):1(TAU) ratio after recruitment onset. With this 2:1-randomization, it became more feasible to start new HOP groups in time before interested individuals were discharged, and therefore the 2:1-randomization was kept until the trial ended. Blinding of participants was not feasible, and research staff were not blinded as outcomes were assessed by self-report. To reduce the risk of contamination between trial arms, HOP participants were asked not to share HOP materials with control group participants.

### Measures of primary outcomes

**Fig. 1:**
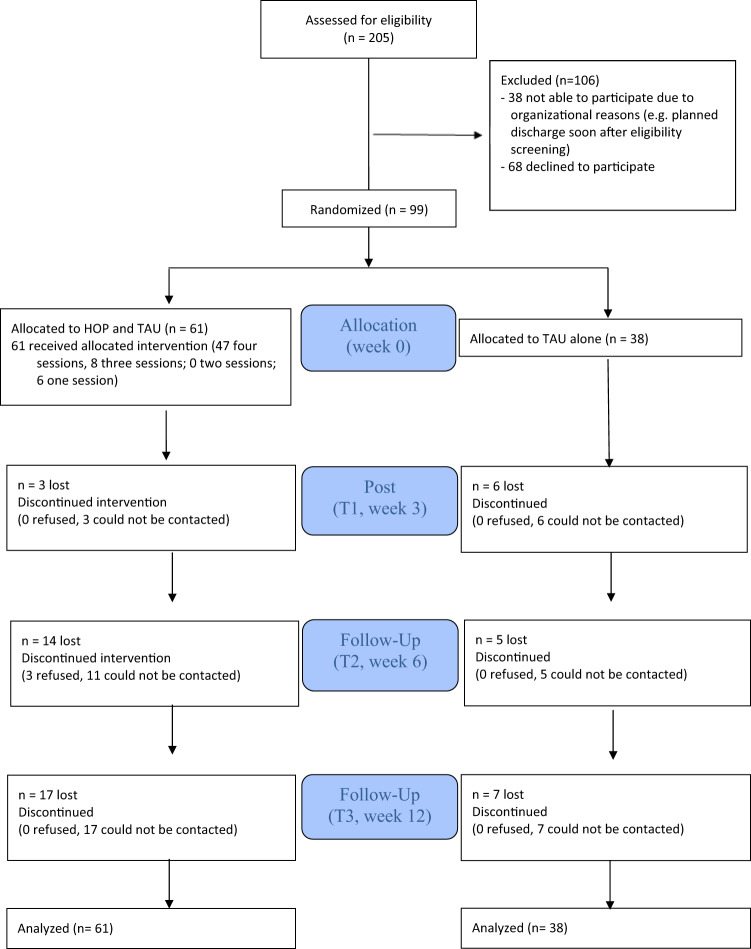
CONSORT flowchart of trial participants

All outcomes were assessed four times (see Fig. 1): at baseline (T0), three weeks later (T1, after lesson 3 for HOP participants), six weeks later (T2, after the booster lesson for HOP participants), and finally 12 weeks after baseline (T3). Based on Lazarus’ stress-coping model [20], our first primary outcome, stigma stress, was measured by the 8-item Stigma Stress Scale that consists of two 4-item subscales [21]: the first on perceived stigma-related harm, with higher mean scores from 1 to 7 indicating more perceived harm (Cronbach’s alphas in this study for T0, T1, T2 and T3, respectively: 0.96/0.94/0.95/0.94), and the second subscale on perceived resources to cope with stigma, again with higher mean scores from 1 to 7 indicating more coping resources (alphas 0.80/0.80/0.88/0.88). Stigma stress is calculated as the difference score of harm minus coping resources, with possible scores from − 6 to + 6 and higher scores equaling more stigma stress. The WHOQOL-BREF is a 26-item measure of quality of life developed by the WHO [22], with higher overall raw mean scores from 1 to 5 indicating better quality of life (alphas 0.90/0.91/0.93/0.93). To make scores comparable to the WHOQOL-100, mean scores are multiplied by 4, resulting in mean scores from 4 to 20. The WHOQOL-BREF has four subscales, among them our second primary outcome at T2, psychological quality of life with 6 items (alphas 0.42/0.57/0.45/0.42).

### Measures of secondary outcomes

Secondary outcomes were assessed by the 9-item Patient Health Questionnaire (PHQ-9 [23], alphas 0.86/0.87/0.89/0.89), with higher sum scores from 0 to 27 indicating more depressive symptoms; the 18-item Psychological Well-Being Scale [24], with higher mean scores from 1 to 6 equalling higher well-being (alphas 0.68/0.63/0.71/0.67); the 9-item self-esteem subscale of the Rogers Empowerment Scale [25], higher mean scores from 1 to 4 reflecting better self-esteem (alphas 0.89/0.92/0.92/0.92); self-stigma with the 10-item version of the Internalized Stigma of Mental Illness Inventory (ISMI-10 [26] with alphas 0.60/0.63/0.61/0.63 and higher mean scores from 1 to 4 equalling more internalized stigma) and with the Self-Stigma of Mental Illness Scale-Short Form (SSMIS-SF [27]) and its 5-item self-concurrence subscale, higher sum scores from 5 to 45 indicating more self-stigma (alphas 0.76/0.70/0.67/0.75). Four items adapted from previous HOP studies [15, 19] assessed attitudes towards disclosure towards a supervisor at work or towards friends and family as well as towards help-seeking through psychotherapy or psychiatric medication. These items were scored from 1 to 7, higher scores reflecting greater comfort with disclosure or help-seeking, respectively. Secrecy was assessed by the five-item secrecy subscale of Bruce Link’s stigma coping orientation scales [28], with higher mean scores from 1 to 6 indicating a stronger tendency to hide one’s mental illness from others (alphas 0.81/0.83/0.84/0.87).

### Analyses

Baseline characteristics of dropouts versus completers at T2, six weeks after baseline, were compared using t-tests or chi-square tests for continuous or binary variables, respectively; the same comparisons were made for baseline variables of HOP versus TAU participants. For the analysis of intervention effects and with two primary endpoints, we corrected the significance level for both endpoints to *p* < 0.025. All other analyses were exploratory with *p* < 0.05. Intervention effects were analyzed with analyses of covariance (ANCOVAs) and all available data (complete case analysis), which is more conservative than a per-protocol analysis because participants who had been randomized to HOP but had not participated in all sessions were included (Fig. 1). Intervention effects were tested using group (HOP versus TAU) as categorical independent variable, the respective outcome (e.g., stigma stress at T1) as dependent variable, and the baseline score of the respective outcome (e.g., stigma stress at T0) as covariate. Effect size estimates are provided as partial η², with 0.01, 0.10 or 0.25 indicating small, medium or large effect sizes, respectively [29]. The qualitative feedback of HOP participants was analyzed using a simple content analysis to identify main themes.

## Results

### Participants

Overall, 99 military servicemembers participated in the trial. Recruitment was challenging; nearly two thirds were randomized to HOP (Fig. 1). More than 80% were male, on average they were in their late 30 s and about a third were single, divorced or separated (Table 1). About one in three participants had a permanent contract with the German military. The most common branch of the military was the army, followed by support and medical services. The majority were non-commissioned officers, with about one quarter each of commanding officers or of enlisted ranks (Table 1). Most participants had received a psychiatric diagnosis about two to three years ago and had been hospitalized about twice before the current inpatient treatment (Table 1). The most common diagnoses were depressive or anxiety disorders as well as posttraumatic stress disorders. There were no significant differences in terms of baseline characteristics between HOP and TAU participants, except for HOP participants having served longer in the German military than TAU participants (T = 2.54, *p* = 0.013; Table 1).

### Comparison of completers versus dropouts

Baseline characterstics of participants may be associated with dropping out of the trial later on. We therefore compared socio-demographic, clinical and disclosure-related baseline variables between completers and dropouts at T2 six weeks after baseline, considering as ‘dropouts’ those participants who did not provide data at T2. Completers did not differ significantly from dropouts in terms of socio-demographic or clinical baseline characteristics (Table 2, all p-values >.2). The only exception was that at baseline dropouts reported more disclosure-related distress than completers at a trend-level (p = 0.05, Table 2).

**Table 1:** Baseline characteristics of 99 trial participants (HOP, *n* = 61, versus TAU/control group, *n* = 38); in the left column see * for significant between-group differences with *p* < 0.05, and (+) for group differences with 0.1 > *p* > 0.05.

		HOP M (SD) or n (%)	TAU/Control M (SD) or *n* (%)
**Socio-demographic variables**			
Age, years		39.4 (9.7)	36.2 (9.9)
Male		50 (82%)	32 (84%)
Single, divorced or separated		19 (31%)	13 (34%)
Have one or more children		38 (62%)	18 (47%)
**Work-related variables**			
Years in the German military*		18.7 (9.6)	13.7 (9.4)
Permanent work contract with the German military		21 (34%)	12 (32%)
Branch of military (missing data for n = 11)	Army	22 (36%)	19 (50%)
	Navy	2 (3%)	2 (5%)
	Air Force	4 (7%)	1 (3%)
	Medical Service	9 (15%)	10 (26%)
	Support Service	14 (23%)	5 (13%)
Rank (missing data for *n* = 1)	Commanding officers	18 (30%)	9 (24%)
	Non-commissioned officers	34 (56%)	18 (47%)
	Enlisted ranks (+)	8 (13%)	11 (29%)
Had at least one deployment abroad		44 (72%)	26 (68%)
**Clinical and diagnostic variables**			
Number of previous psychiatric inpatient treatments (before the current one, if applicable; missing data for *n* = 7): mean (SD)/median/range		2.5 (2.6)/ 2/0 to 10	3.0 (2.7)/ 2/0 to 9
Years since first psychiatric diagnosis, missing data for *n* = 8: mean (SD)/median/range (+)		3.4 (3.5)/ 2/0 to 12	4.8 (4.1)/ 3/0 to 16
Depressive disorder		41 (67%)	25 (66%)
Anxiety disorder		21 (34%)	16 (42%)
Posttraumatic stress disorder		40 (66%)	25 (66%)
Alcohol or substance use disorder		7 (12%)	5 (13%)

**Table 2:** Baseline/T0 characteristics of completers vs. dropouts (‘dropout’ referring to participants who did not complete the T2 assessment)

	Completers (n = 80) M (SD) or *n* (%)	Dropouts (*n* = 19) M (SD) or *n* (%)	T	χ^2^	*p*
Socio-demographic variables					
Age, years	38.6 (9.9)	36.5 (10.3)	−0.84		0.40
Male	66 (83%)	16 (84%)		0.25	0.89
Single, divorced or separated	25 (33%)	7 (39%)		0.27	0.60
Permanent work contract	29 (36%)	4 (21%)		1.60	0.21
Clinical variables					
Depressive symptoms^a^	13.4 (5.5)	13.4 (7.0)	0.04		0.97
Number of psychiatric inpatient treatments (before the current one, if applicable)	2.8 (2.6)	2.3 (2.6)	0.73		0.47
Years since first psych. diagnosis	4.1 (3.7)	3.6 (4.1)	−0.41		0.68
Depressive disorder	55 (69%)	11 (58%)		0.81	0.37
Anxiety disorder	32 (40%)	5 (26%)		1.23	0.27
Posttraumatic stress disorder	54 (68%)	11 (58%)		1.26	0.26
Alcohol or substance use disorder	10 (13%)	2 (11%)		0.06	0.81
Disclosure variables					
Attitudes to disclosure towards supervisor at work (from 1/not at all comfortable to 7/very comfortable)	3.1 (1.7)	3.2 (1.7)	0.31		0.76
Attitudes to disclosure towards family/friends	4.2 (1.9)	4.0 (1.9)	−0.34		0.74
Disclosure-related distress^b^	4.2 (1.7)	5.1 (1.5)	2.00		0.05

^a^Patient Health Questionnaire (PHQ-9 [23])

^b^‘In general, how distressed or worried are you with respect to secrecy or disclosure of your mental illness to others?’, rated from 1/not at all to 7/very much

### The two primary endpoints

With regard to our first primary endpoint, HOP participants showed significantly lower stigma stress at T1 (3 weeks after baseline) compared to the control group, with a medium effect size (Table 3 in bold print). Our second primary endpoint, psychological quality of life at T2 (6 weeks after baseline and after the HOP booster session for HOP participants), indicated only a small effect with a non-significant increase among HOP participants compared to the control group (Table 3 in bold print).

**Table 3:** ANCOVAs for HOP and TAU groups for stigma stress and quality of life (with baseline/T0 score of each outcome variable as covariate), both primary endpoints in bold. T1 is 3 weeks after baseline, T2 is 6 weeks after baseline, T3 is 12 weeks after baseline

Outcomes		T0 M (SE)	T1 M (SE)	T1 group difference M (95%-CI)	T2 M (SE)	T2 group difference M (95%-CI)	T3 M (SE)	T3 group difference M (95%-CI)	T1			T2			T3		
									F	η^2^	*p*	F	η^2^	*p*	F	η^2^	*p*
Stigma stress^a^	HOP	−0.68 (0.36)	**−2.00 (0.23)**	**−1.32**	−2.37 (0.23)	−1.87	−2.23 (0.27)	−1.33	**11.7**	**0.12**	**< 0.001**	19.6	0.20	< 0.001	10.0	0.12	0.002
	TAU	−0.34 (0.39)	**−0.67 (0.31)**	**(−2.09 to −0.55)**	−0.50 (0.31)	(−2.72 to −1.03)	−0.90 (0.32)	(−2.16 to −0.49)									
Overall quality of life^b^	HOP	12.62 (0.28)	12.97 (0.13)	0.18	13.36 (0.13)	0.71	13.20 (0.20)	0.61	0.7	0.01	0.42	5.8	0.07	0.02	3.8	0.05	0.055
	TAU	12.75 (0.29)	12.79 (0.18)	(−0.26 to 0.61)	12.65 (0.18)	(0.12 to 1.28)	12.58 (0.24)	(−0.01 to 1.24)									
Psychological quality of life^b^	HOP	11.44 (0.28)	11.88 (0.19)	−0.18	**12.37 (0.19)**	**0.47**	12.01 (0.24)	0.59	0.3	0.0	0.58	**1.6**	**0.02**	**0.21**	2.5	0.03	0.12
	TAU	12.09 (0.31)	12.06 (0.26)	(−0.83 to 0.46)	**11.90 (0.26)**	**(−0.27 to 1.21)**	11.42 (0.28)	(−0.15 to 1.32)									

^a^ Stigma Stress Scale [21]; ^b^ WHQOOL-BREF [22]

**Table 4:** ANCOVAs for HOP and TAU groups and secondary outcomes (with baseline/T0 score of each outcome variable as covariate). T1 is 3 weeks after baseline, T2 is 6 weeks after baseline, T3 is 12 weeks after baseline

Outcomes		T0 M (SE)	T1 M (SE)	T1 group difference M (95%-CI)	T2 M (SE)	T2 group difference M (95%-CI)	T3 M (SE)	T3 group difference M (95%-CI)	T1			T2			T3		
									F	η^2^	*p*	F	η^2^	*p*	F	η^2^	*p*
Self-stigma (ISMI-SF)^a^	HOP	2.38 (0.08)	2.24 (0.05)	−0.16	2.10 (0.05)	−0.31	2.16 (0.05)	−0.12	4.3	0.05	0.04	10.7	0.12	0.002	2.1	0.03	0.15
	TAU	2.39 (0.09)	2.40 (0.06)	(−0.31 to −0.01)	2.41 (0.06)	(−0.50 to −0.12)	2.28 (0.06)	(−0.29 to 0.05)									
Self-stigma (SSMIS-SF)^b^	HOP	17.61 (1.12)	17.64 (0.57)	0.78	15.95 (0.57)	−2.11	16.52 (0.96)	0.26	0.7	0.01	0.42	2.5	0.03	0.12	0.0	0.00	0.86
	TAU	18.32 (1.36)	16.87 (0.77)	(−1.14 to 2.69)	18.06 (0.77)	(−4.79 to 0.57)	16.26 (1.14)	(−2.70 to 3.22)									
Depressive symptoms^c^	HOP	13.59 (0.78)	11.58 (0.51)	−0.95	10.84 (0.51)	−2.00	10.43 (0.57)	−1.44	1.3	0.01	0.27	5.1	0.06	0.03	2.6	0.04	0.11
	TAU	13.03 (0.85)	12.54 (0.68)	(−2.64 to 0.74)	12.84 (0.68)	(−3.77 to −0.23)	11.87 (0.68)	(−3.20 to 0.33)									
Empowerment^d^	HOP	2.76 (0.08)	2.90 (0.05)	0.08	3.02 (0.05)	0.28	2.96 (0.05)	0.06	0.8	0.01	0.36	9.2	0.11	0.003	0.4	0.01	0.51
	TAU	2.78 (0.09)	2.82 (0.07)	(−0.09 to 0.24)	2.74 (0.07)	(0.10 to 0.47)	2.90 (0.06)	(−0.11 to 0.22)									
Well-being^e^	HOP	3.98 (0.09)	4.05 (0.04)	0.10	4.21 (0.04)	0.23	4.16 (0.05)	0.04	1.9	0.02	0.17	5.8	0.07	0.02	0.3	0.0	0.60
	TAU	4.04 (0.10)	3.95 (0.06)	(−0.05 to 0.25)	3.97 (0.06)	(0.04 to 0.42)	4.12 (0.06)	(−0.12 to 0.20)									
Disclosure-related distress^f^	HOP	4.26 (0.24)	3.99 (0.16)	−0.54	3.61 (0.16)	−1.03	3.68 (0.25)	−0.58	1.9	0.02	0.17	9.4	0.11	0.003	2.1	0.03	0.15
	TAU	4.55 (0.23)	4.37 (0.22)	(−1.26 to 0.17)	4.64 (0.22)	(−1.70 to −0.36)	4.26 (0.30)	(−1.37 to 0.21)									
Attitude to disclosure (family/friends)	HOP	4.26 (0.24)	4.53 (0.19)	0.40	4.69 (0.19)	0.76	4.54 (0.23)	0.19	1.7	0.02	0.20	5.3	0.07	0.02	0.3	0.0	0.61
	TAU	3.92 (0.30)	4.13 (0.25)	(−0.21 to 1.03)	3.93 (0.25)	(0.10 to 1.42)	4.36 (0.28)	(−0.55 to 0.92)									
Attitude to dis-closure (supervisor at work)	HOP	3.42 (0.23)	3.52 (0.20)	0.64	3.68 (0.20)	0.77	3.88 (0.26)	0.84	3.7	0.04	0.06	4.8	0.06	0.03	4.3	0.06	0.04
	TAU	2.74 (0.23)	2.88 (0.26)	(−0.02 to 1.30)	2.91 (0.26)	(0.07 to 1.48)	3.04 (0.31)	(0.03 to 1.65)									
Attitude to psychotherapy	HOP	6.57 (0.09)	6.50 (0.10)	−0.12	6.77 (0.10)	0.34	6.64 (0.11)	0.12	0.6	0.01	0.45	4.2	0.05	0.04	0.5	0.01	0.47
	TAU	6.53 (0.12)	6.62 (0.13)	(−0.45 to 0.20)	6.42 (0.13)	(0.01 to 0.67)	6.52 (0.13)	(−0.21 to 0.45)									
Secrecy^g^	HOP	3.68 (0.14)	3.48 (0.10)	−0.38	3.32 (0.17)	−0.76	3.43 (0.12)	−0.51	5.2	0.06	0.03	13.8	0.15	< 0.001	7.0	0.09	0.01
	TAU	3.78 (0.19)	3.85 (0.13)	(−0.70 to −0.05)	4.09(0.17)	(−1.17 to −0.35)	3.94 (0.15)	(−0.90 to −0.13)									

^a^ Internalized Stigma of Mental Illness Inventory, Short Form [26]; ^b^ Self-Stigma of Mental Illness Scale-Short Form, subscale apply/self-concurrence [27]; ^c^ PHQ-9 [23]; ^d^ Empowerment Scale [25]; ^e^ Psychological Well-Being Scale [24]; ^f^ Disclosure Distress [19]; ^g^ Stigma Coping Orientation Scales, Secrecy Scale [28]

### Secondary outcomes

All other outcomes (all at T1 except stigma stress, all at T2 except psychological quality of life, all at T3) were considered secondary. At T1, there were significant positive effects of HOP on self-stigma as assessed by the ISMI (not as measured by the SSMIS-SF) and for secrecy, both with small-to-medium effect sizes. There was a trend-level positive effect on attitudes towards disclosing one’s mental illness to a supervisor at work (Table 4). At T2, there were significant positive effects for almost all secondary outcomes with mostly medium effect sizes. The effect on stigma stress increased to a large effect size at T2, and the effect on overall quality of life was significant at T2 with a small-to-medium effect size (Table 4). At the T3 follow-up assessment (12 weeks after baseline), only HOP effects on stigma stress, on disclosure attitudes at work and on secrecy remained significant, with effects on overall quality of life remaining marginally significant (*p* = 0.055, Table 4).

### Participants’ views

Participants of all HOP groups perceived the fact that groups were facilitated by peers (military servicemembers with a history of mental illness who had been trained in HOP) as very positive since participants felt understood and did not have to explain personal challenges. Instead they and the group facilitators had a shared understanding of the consequences of mental disorders for military personnel. Participants also welcomed the safe environment in the HOP groups with the other peers; however, some participants felt uncomfortable if they knew others in the same HOP group from their workplace. Both participants and peer group facilitators alike felt that the two hours for each group session, as standardized in the study protocol, were at times too short to cover all aspects of the HOP workbook in detail, especially in the first session, limiting interactions between group members. The interval between the four lessons, including the three weeks between lesson three and the fourth booster session, on the other hand, appeared ideal because participants could review material between sessions and gain experience with (non)-disclosure. In summary, HOP was seen as very helpful to reduce the impact of mental illness stigma for military servicemembers. In the view of participants and group facilitators alike, HOP should be implemented in the German military’s routine care; ideally with more time per session, but with the same intervals between sessions as in the current project.

## Discussion

This study provides first evidence on the feasibility and efficacy of HOP for active military personnel. To the best of our knowledge, HOP had not been evaluated in this group. Despite recruitment difficulties, it proved feasible to train military servicemembers with lived experience of mental illness as group facilitators, to maintain high manual fidelity throughout the trial, and to keep participants in the trial with 90% of all HOP participants attending at least three out of four HOP sessions.

The findings supported our first hypothesis that HOP would reduce stigma stress after the third HOP lesson (T1). On the other hand, our second hypothesis about HOP effects on psychological quality of life at T2 was not confirmed; this may reflect a lack of efficacy regarding psychological quality of life or may be caused by the very low reliability of this subscale (Cronbach’s alphas ≤ 0.45 at T0 and at T2). The latter interpretation is consistent with significant positive effects of HOP on overall quality of life at T2 and much higher reliability of the overall quality of life scale (alphas ≥ 0.9 at T0 and T2). The fact that HOP, despite its brevity with four two-hour sessions, significantly improved overall quality of life is encouraging.

Regarding secondary outcomes, there were few significant positive HOP effects at T1, but we found positive effects in most domains at T2. As the HOP booster session took place prior to the T2 measurement, this suggests the booster was needed for HOP to be effective; however, a trial comparing HOP with versus without booster would be necessary to answer this question. The fact that self-stigma showed HOP benefits at T1 and T2, but only as assessed by the ISMI-10 and not by the SSMIS-SF, is a topic for future research. Possibly the ISMI is more sensitive to change as its items, unlike the SSIMS-SF, cover a broad mixture of agreement with negative stereotypes, perceived devaluation-discrimination, social withdrawal and self-stigma in a strict sense: application of negative stereotypes to oneself as measured by the SSMIS-SF. The latter may be more difficult to change by a brief intervention like HOP.

### Implications for practice & anti-stigma programs in the military

The results of our trial as well as the feedback of HOP peer group facilitators and participants suggest that a broader implementation of HOP in the German military would be welcome in the view of HOP participants. Following a future confirmatory trial in different military settings, it could be decided in which settings HOP is most helpful. Ideally, programs such as HOP should be accompanied by programs to improve attitudes of military servicemembers without mental illness. Otherwise, military personnel with mental illness alone would carry the burden of coping with stigma, but stigma reduction is a task for the whole organization [11].

The HOP program could play a significant role in destigmatizing mental health issues in the military which often maintains a strong stigma surrounding mental illness. Military servicemembers are often reluctant to disclose mental health issues for fear of being perceived as weak [4, 13]. Introducing a program like HOP could help military personnel to talk about their mental health, if they decide to do so. A key consideration in implementing HOP in the military is cultural sensitivity and ensuring that the program is presented in a way that promotes acceptance of mental health issues, while also ensuring that participants do not view seeking help as a sign of weakness. Potential positive outcomes include increased utilization of psychosocial services since previous studies highlighted low rates of help-seeking in the German military [4].

To achieve a broader impact, a contact-based program for all military personnel to improve attitudes towards colleagues with mental health issues should accompany HOP and be integrated into the first Basic Military Training. In this context, social contact refers to cooperative, friendly contact, e.g. in a workshop, between military staff with and without mental illness that is supported by military leaders [30, 31]. This would help to promote an open communication culture and facilitate access to HOP later on, if and when military personnel develop mental health problems and struggle with disclosure decisions. The interactions between such a contact-based program and HOP in the military is a topic for future research and was not examined in this study.

As military leaders play a crucial role in the perception and management of mental health issues, they should be trained to recognize signs of mental health challenges to establish a supportive and open attitude. Finally, in our study a number of participants felt uncomfortable when other participants were known to them, for example because they had served in the same unit. Therefore, to ensure that military personnel are not discouraged from participating in the program due to potential negative consequences or discomfort, privacy protections are important.

### Implications for research

Given the encouraging findings of this pilot trial, a future project should examine HOP and its effectiveness among active military servicemembers in a larger and more diverse sample in terms of urban and rural regions and by including a larger proportion of outpatients and including personnel who are no longer in mental health care. A longer follow-up period would be desirable especially regarding more distal potential HOP outcomes such as actual help-seeking or vocational functioning. Subsequent studies could compare HOP for the military with HOP for veterans or with HOP for first responders such as police officers who share many characteristics with military personnel [32].

### Limitations

Several limitations of this study need to be considered. First, we assessed manual fidelity throughout, but the long trial period may have led to fluctuations in HOP delivery. Second, recruitment was slow which echoes the experience in previous HOP trials that most participants enjoy HOP after entering the program, but it can be difficult to motivate people to join initially [15]. In our case, recruitment became more difficult due to external factors, including the unexpected closure of a psychiatric day clinic and legal access restrictions during the COVID-19 pandemic. Additionally, the start of new HOP groups required a minimum number of participants, which was not always feasible within the treatment timeframes. Third, the randomization ratio was adjusted from 1:1 to 2(HOP and TAU):1(TAU alone) during the trial to prevent dropout of participants waiting for the start of a new HOP group. Fourth, participants and study staff were not blinded, given the open, group-based nature of the HOP intervention and the use of self-report measures only. Fifth, we did not record the exact therapeutic modalities received within TAU across groups which limits conclusions about potential interactions between HOP and TAU; that said, in Germany outpatient guideline-based psychotherapy typically consists of one 50-minute session per week, while inpatient and day clinic programs involve multimodal interventions (see above in the Methods for details). Sixth, participants were recruited from a single military mental health center and sample characteristics (predominantly middle-aged male soldiers) restrict generalizability. Seventh, dropout rates were moderate, but a substantial part of attrition can be attributed to the Covid-19 pandemic: entire group cycles had to be cancelled due to legal access restrictions (e.g., vaccination or recovery status), which some potential participants could not meet, or group sessions had to be rescheduled due to pandemic-related circumstances and some participants were unable to attend on alternative dates. Other participants discontinued due to military obligations, such as deployments abroad. Eighth, 68 individuals declined to participate in the trial (Fig. 1). Although formal reasons for refusal were not collected, our impression was that stigma-related concerns contributed to their decision. Finally, there was no active control condition, e.g. a psychoeducation group or simple peer support, to rule out non-specific intervention effects.

## Conclusions

This first trial of HOP among active military servicemembers provides encouraging first evidence that HOP may be a feasible and helpful intervention for military personnel with mental illness in terms of reductions in stigma stress, self-stigma, depressive symptoms and increased well-being, empowerment and quality of life. Future trials should replicate our findings in different settings and in other military as well as veteran populations before a wider implementation can be discussed. Future research should also examine whether these effects translate into changes in help-seeking and improved social and vocational functioning over time.

## Data Availability

Research data is not accessible.
